# Effectiveness of wound infusion of 0.2% ropivacaine by patient control analgesia pump after minithoracotomy aortic valve replacement: a randomized, double-blind, placebo-controlled trial

**DOI:** 10.1186/s12871-020-01093-9

**Published:** 2020-07-18

**Authors:** Gordan Mijovski, Matej Podbregar, Juš Kšela, Matej Jenko, Maja Šoštarič

**Affiliations:** 1 Department of Anaesthesiology and Surgical Intensive Therapy, University Medical Centre Ljubljana, Faculty of Medicine, University of Ljubljana, Zaloška cesta 2, 1000 Ljubljana, Slovenia; 2 Department of Cardiovascular Surgery, University Medical Centre Ljubljana, Faculty of Medicine, University of Ljubljana, Ljubljana, Slovenia

**Keywords:** Wound catheter, PCA, AVR, Minithoracotomy, Multimodal analgesia

## Abstract

**Background:**

Local anesthetic wound infusion has become an invaluable technique in multimodal analgesia. The effectiveness of wound infusion of 0.2% ropivacaine delivered by patient controlled analgesia (PCA) pump has not been evaluated in minimally invasive cardiac surgery. We tested the hypothesis that 0.2% ropivacaine wound infusion by PCA pump reduces the cumulative dose of opioid needed in the first 48 h after minithoracothomy aortic valve replacement (AVR).

**Methods:**

In this prospective, randomized, double-blind, placebo-controlled study, 70 adult patients (31 female and 39 male) were analyzed. Patients were randomized to receive 0.2% ropivacaine or 0.9% saline wound infusion by PCA pump for 48 h postoperatively. PCA pump was programmed at 5 ml h^− 1^ continuously and 5 ml of bolus with 60 min lockout. Pain levels were assessed and recorded hourly by Numeric Rating Scale (NRS). If NRS score was higher than three the patient was administered 3 mg of opioid piritramide repeated and titrated as needed until pain relief was achieved. The primary outcome was the cumulative dose of the opioid piritramide in the first 48 h after surgery. Secondary outcomes were frequency of NRS scores higher than three, patient’s satisfaction with pain relief, hospital length of stay, side effects related to the local anesthetic and complications related to the wound catheter.

**Results:**

The cumulative dose of the opioid piritramide in the first 48 h after minithoracotomy AVR was significantly lower (*p* < 0.001) in the ropivacaine (R) group median 3 mg (IQR 6 mg) vs. 9 mg (IQR 9 mg). The number of episodes of pain where NRS score was greater than three median 2 (IQR 2), vs 3 (IQR 3), (*p* = 0.002) in the first 48 h after surgery were significantly lower in the ropivacaine group, compared to control. Patient satisfaction with pain relief in our study was high. There were no wound infections and no side-effects from the local anesthetic.

**Conclusions:**

Wound infusion of local anesthetic by PCA pump significantly reduced opioid dose needed and improves pain control postoperatively. We have also shown that it is a feasible method of analgesia and it should be considered in the multimodal pain control strategy following minimally invasive cardiac surgery.

**Trial registration:**

ClinicalTrials.gov NCT03079830, date of registration: March 15, 2017. Retrospecitvely registered.

## Background

Multimodality in pain management, during and after surgery, has long been established and well accepted [1]. Since, it has become a central part of most enhanced recovery protocols and its use has received high evidence level and strong recommendation [2, 3]. Despite the wide acceptance of the concept and the ever greater focus on postoperative pain relief and fast tracking of patients there are numerous reports of suboptimal pain management [4, 5]. While wound infusion catheter is becoming more and more popular way of pain relief management after surgery [6], opioids are still a mainstream medication for pain relief after cardiac surgery. The concept of multimodal analgesia implies combining medications with different mechanisms of action to achieve effective postoperative pain relief while avoiding their adverse effects, mainly those of opioids [7]. The most frequent side effects of opioid analgesics being respiratory depression, nausea, constipation and pruritus [8–11]. To avoid these side effects of opioid medications postoperatively, they are often combined with NSAIDs in cardiac surgery [12–14]. Still, opioids have remained the main analgesia of choice following cardiac surgery in the early postoperative period and serve as a reference point to which most analgesic protocols are compared to.

Delivering local anesthetic through a wound catheter was proven to be a successful way of postoperative pain relief throughout most surgical fields [15–17]. In cardiac surgery however, it has produced mixed results when used after full sternotomy [18–21]. The increasing use of minimally invasive surgical techniques in heart surgery offers more opportunities for successfully implementing multimodality by administering local anesthetic through a wound catheter. The effectiveness of wound infusion of 0.2% ropivacaine delivered by patient controlled analgesia (PCA) pump has not been evaluated in minimally invasive cardiac surgery.

We designed a prospective, randomized, double-blind, placebo-controlled trial, to analyze the effectiveness of wound infusion of 0.2% ropivacaine delivered by patient control analgesia (PCA) pump for pain relief after minimally invasive right anterior minithoracotomy aortic valve replacement (AVR).

## Methods

This study was approved by the National Medical Ethics Committee of Republic of Slovenia (No. MZ 0120–145/2016–3, 10.06.2016) and registered at ClinicalTrials.gov (NCT03079830). The study was conducted in a tertiary level university hospital from March 2017 to January 2018. With this prospective, randomized, double-blind, placebo-controlled study we analyzed the effectiveness of wound infusion of local anesthtetic 0,2% ropivacaine after minimally invasive - right anterior minithoracotomy AVR. The study adheres to the CONSORT guidelines for reporting research.

All patients were preoperatively given a detailed description of the study by an anesthetist. After obtaining written consent, the patients were familiarized with the numeric rating scale (NRS) for pain evaluation where “0” represents no pain and “10” represents worst possible pain.

The protocol we describe was a result of a small pilot study we carried out before the main study. Inclusion criteria were all adult patients scheduled to have an elective right anterior minithoracotomy AVR who consented to be included in the study. Preoperative exclusion criteria were patients not consenting to the study, emergency surgery, patients allergic to local anesthetic and patients with chronic pain syndromes. Postoperative exclusion criteria were reoperation in the first 48 h after surgery and prolonged need for intubation postoperatively. In total 76 adult patients scheduled to have an elective right anterior minithoracotomy AVR were randomly allocated into two groups. All patients were operated by the same surgical team and all patients received the same sutureless aortic valve (Perceval - LivaNova PLC, London, UK).

All patients were premedicated one hour before the surgery with 5 mg diazepam orally. Fentanyl 5–10 μg kg^− 1^, ethomidate 0.2 mg kg^− 1^ were used as induction agents and rocuronium 0.6 mg kg^− 1^ was used as neuromuscular blocking drug. Intubation was performed with a single lumen tube. Total intravenous anesthesia was maintained with 0.3 μg kg^− 1^ min^− 1^ remifentanyl and 5 mg kg^− 1^ h^− 1^ propofol. Standard haemodynamic monitoring for cardiac surgery was used during the procedure. Our standard monitoring includes direct arterial blood pressure, central venous pressure, transoesophageal echocardiography, body temperature, urinary catheter and cerebral oximeter.

**Fig. 1:**
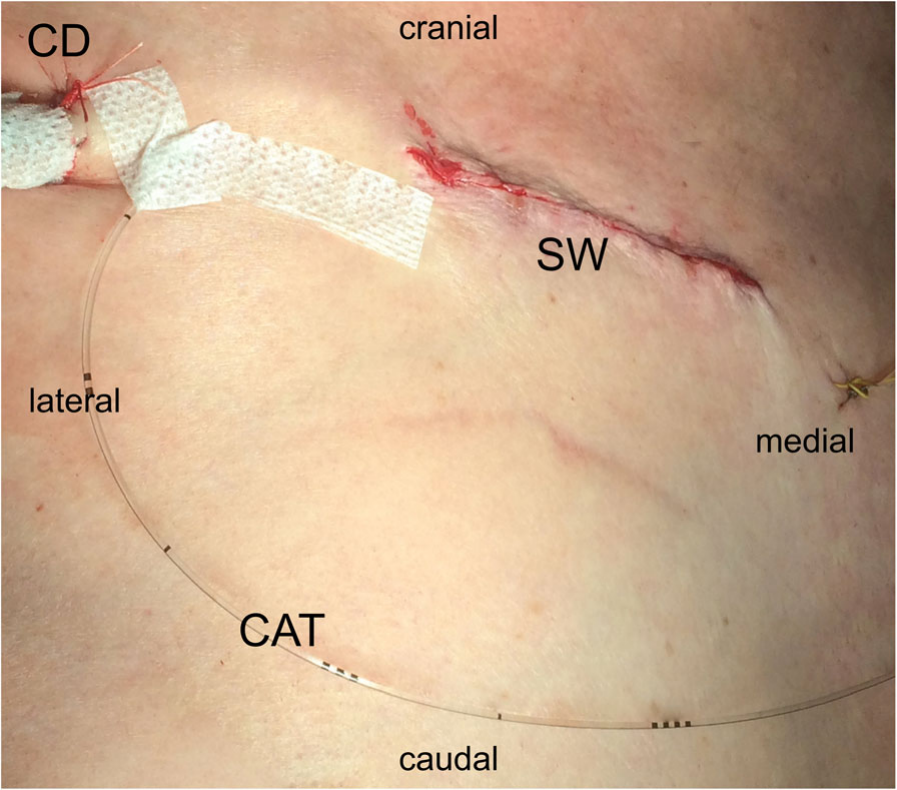
Wound catheter position. CAT-wound catheter, CD-chest drain, SW-surgical wound

At the end of the operation after wound closure, the surgeon inserted a 7.5 cm long wound catheter (PAINfusor - Plan 1 Health Srl, Amaro UD, Italy) by using an introducer needle. The catheter was placed above the ribs in the deep subcutaneous tissue followed by a bolus of 10 ml of 0.75% ropivacaine through the catheter (Fig. 1). We administered a bolus of 0.75% ropivacaine to patients of both groups at the end of the operation, regardless of the group they were randomized to as we were using an ultra short-acting opioid for maintaining analgesia perioperatively. A PCA pump (Mini Rythmic Evolution, Micrel Medical Devices SA - Athens, Greece) was connected to the catheter and started in theatre at 5 ml h^− 1^. Before stopping the remifentanil infusion at the end of the operation all patients were administered 2.5 g i.v. metamizole, a non-opioid analgesic. After the operation patients were admitted to the ICU. Once patients met the standard extubation protocol requirements they were extubated (Tab1). The feasibility of intervention was evaluated in a pilot study of 20 patients, as part of protocol development of the RCT.

**Table 1:** Extubation criteria in the ICU

Parameter	Values
Consciousness	Alert, obeys simple commands
Ventilation	Spontaneous, respiratory rate 10–18/min, TV ≥ 6 ml/kg, SaO2 ≥ 94% on FiO2 ≤ 0.35
Haemodynamics	MAP 60–80 mmHg, heart rate 50–90 beats/min, no signs of myocardial ischemia nor vasoplegia
Bleeding	≤ 2 ml/kg/h in first two hours
Body temperature	Between 37 °C–36 °C

MAP - mean arterial pressurre; TV - tidal volume

The patients were randomized into two groups: Study group - R (ropivacaine) - 38 patients and Control group - C (0.9% saline) - 38 patients. A dedicated nurse that was not part of the performing anesthetic team was in charge of the randomization process. Randomization was done by covariate adaptive randomization using an on-line program for randomization at www.graphpad.com. The dedicated nurse who was the only person to know which group the patient was randomized to, prepared the mixtures. The bags containing both mixtures were of same shape, taped and covered with aluminum foil.

The anesthetist in the operating theatre, the intensivists and the nurses in the ICU and HDU were all blinded to which group a patient was randomized to. The PCA pump was programmed the same way for both groups, to continuously administer the mixture at 5 ml h^− 1^ and a bolus of 5 ml if needed with a lock out time of 60 min.

1. Patients in the ropivacaine group were administered 5 ml h^− 1^ of 0.2% ropivacaine continuously per PCA pump through the wound catheter and a single 5 ml bolus if needed with 60 min of lock-out time.

2. Patients in the 0,9% saline group were administered 5 ml h^− 1^ of 0.9% saline continuously per PCA pump through the wound catheter and a single 5 ml bolus if needed with 60 min of lock-out time.

Postoperatively in the ICU, patients in both groups regularly received metamizole 2,5 g/12 h i.v. Once the patient was awake, extubated and able to communicate, the ICU nurse hourly assessed and recorded the pain level by NRS score, except when patients were asleep. If the pain level was higher than three, the patient at first administered a bolus of 5 ml of the mixture delivered by the PCA pump through the wound catheter. If the NRS pain score remained higher than three, 15 min after the PCA bolus, the patient was administered i.v. bolus of 3 mg piritramide - an opioid 0.75 times as potent as morphine [22], by a nurse or physician, repeated and titrated as needed until pain relief was achieved. The wound infusion was administered during the first 48 h after surgery when the catheter was removed. The patients were continuously clinically assessed for side effects related to local anesthetic - neurotoxicity and cardiotoxicity, and complications related to the wound catheter - wound infection or delayed healing.

The dedicated nurse using Stratified Randomization randomly selected 20 patients to have total plasma ropivacaine concentration measured (ten from each group). Also 20 patients (ten from each group) were randomly selected, using the same method, to have the tips of wound catheters’ sent for microbiology analysis after removal. Venous blood samples for total plasma ropivacaine concentration were taken at 1 h, 24 h and 48 h after surgery. The blood samples were centrifuged immediately at 3000 rpm for 5 min and plasma was aspirated and pipetted into a separate tube. The tubes with the plasma samples were then frozen at − 60**°** and analyzed after all the samples from all 20 patients were taken.

The primary outcome of our study was the cumulative dose of the opioid piritramide required in the 48 h after surgery, compared between the two groups. Our secondary outcomes were the frequency of NRS scores higher than three, patients’ satisfaction with the pain relief, the time to recovery and discharge from hospital, side effects related to local anesthetic and complications related to the wound catheter. Patients’ satisfaction with the pain relief was assessed on the third postoperative day by anesthetist trainees, that were blinded of the treatment allocation, on a patient satisfaction scale with the possible answers ranging from 1.completely satisfied, 2.satisfied, 3.neither satisfied nor dissatisfied, 4.dissatisfied, to 5.completely dissatisfied.

### Statistical analysis

Before carrying out the main study, we performed a small pilot study with 20 patients, to assess the feasibility of the method and for sample size calculations. Results are based on the primary outcome, piritramide consumption in the first 48 h after surgery. To achieve 85% of statistical power at least 35 patients per group had to be included into the study. Effect size for our calculaction was 1.09. Groups of patients were compared by Mann-Whitney U test or χ2 test where appropriate. P -value below 0.05 was used for statistical significance. Statistical analysis was performed using the R project, a language and environment for statistical computing - R Foundation for Statistical Computing, Vienna, Austria.

## Results

**Fig. 2:**
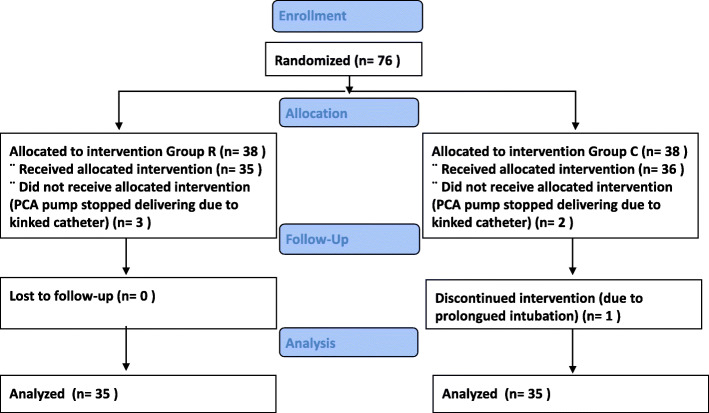
CONSORT flow diagram of study inclusion

Overall, 76 eligible patients consented to participate in the study. Postoperatively 3 patients from the ropivacaine group and 2 patients from the 0.9% saline group were excluded due to occlusion of the wound catheter. Also, one patient from the 0.9% saline group was excluded due to prolonged need for intubation postoperatively, not related to the wound catheter. Thirty-five patients in each group completed the study and their results were analyzed (Fig. 2). Patients in both groups had similar baseline demographic (Tab. 2) and clinical characteristics (Tab. 3).

**Table 2:** Demographic data for both groups

Demographic data	Ropivacaine group	0,9% saline group	*P* value
No. of patients studied	35	35	
Age, median (25th–75th percentilles) in years	76 (69–78)	76 (72–81)	0.083^1^
Female	15	16	0.810^2^
Male	20	19	
No. of patients smoking	7	6	0.758^2^
*1 – Mann-Whitney U test 2 – χ*^*2*^*test*

**Table 3:** Clinical data for both groups

Clinical data median (25th–75th percentilles)	Ropivacaine group	0,9% saline group	P value
Euroscore II	1.35 (0.99–2.01)	1.56 (1.17–2.15)	0.173^1^
CPB time in minutes	60 (54–69)	66 (58–77)	***0.047***^***1,3***^
Cross clamp time in minutes	33 (30–37)	36 (32–45)	0.057^1^
Time of surgery in minutes	136 (122–145)	144 (132–154)	0.101^1^
Time to extubation in minutes	120 (120–180)	120 (120–180)	0.703^1^
PCA boluses attempted	2 (1–4)	4 (2–6)	***0.022***^***1,3***^
PCA boluses given	2 (1–4)	3 (2–5)	***0.021***^***1,3***^
No. of patients with PONV	7	8	0.778^2^
1 – Mann-Whitney U test, 2 – χ^2^ test 3-statistically significant difference			

**Fig. 3:**
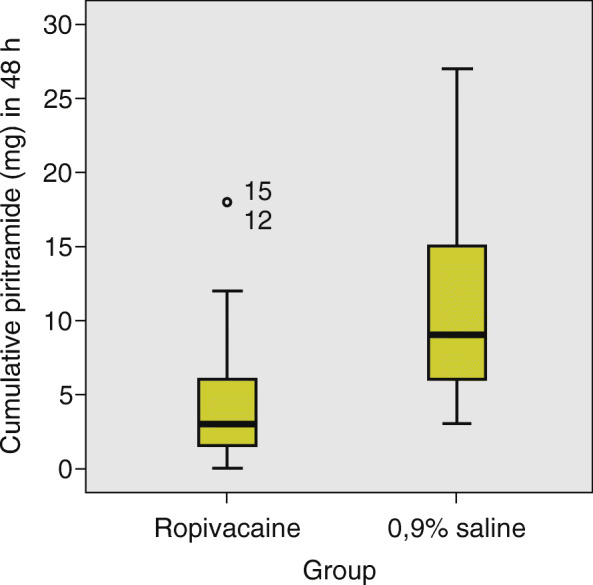
Comparison of cumulative dose of piritramide in the first 48 h postoperatively between the ropivacaine group and the 0.9% saline group

The primary outcome of the cumulative dose of the opioid piritramide in the first 48 h after minithoracotomy AVR was significantly lower (*p* < 0.001, Mann-Whitney U test) in the ropivacaine group. The median cumulative dose of piritramide in 48 h in the ropivacaine group was 3 mg (IQR 6 mg). The median dose of piritramide in the 0.9% saline group was 9 mg (IQR 9 mg). We found that the difference between the two groups was statistically significant (p < 0.001), (Fig. 3).

**Fig. 4:**
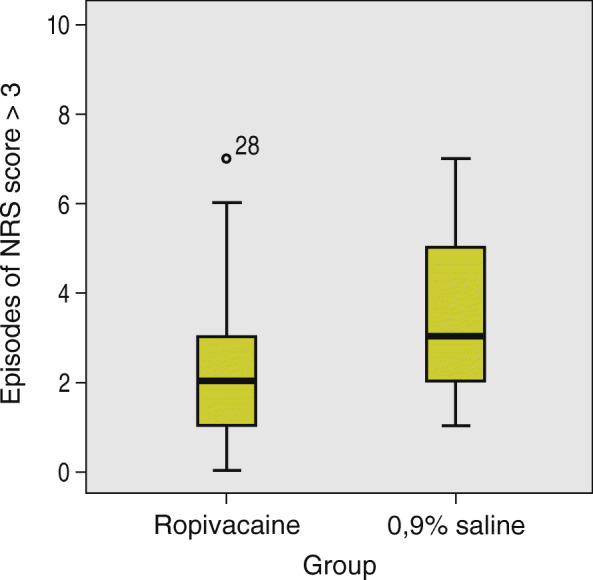
Comparison of number of episodes of NRS score higher than three in the first 48 h postoperatively between the ropivacaine group and the 0,9% saline group

Among the secondary outcomes, only the frequency of NRS scores higher than three in the first 48 h postoperatively reached statistical significance (*p* = 0.002). The median number of episodes of pain where NRS score was greater than three in the ropivacaine group was 2 (IQR2). The median in the 0.9% saline group was 3 (IQR 3) (Fig. 4).

**Fig. 5:**
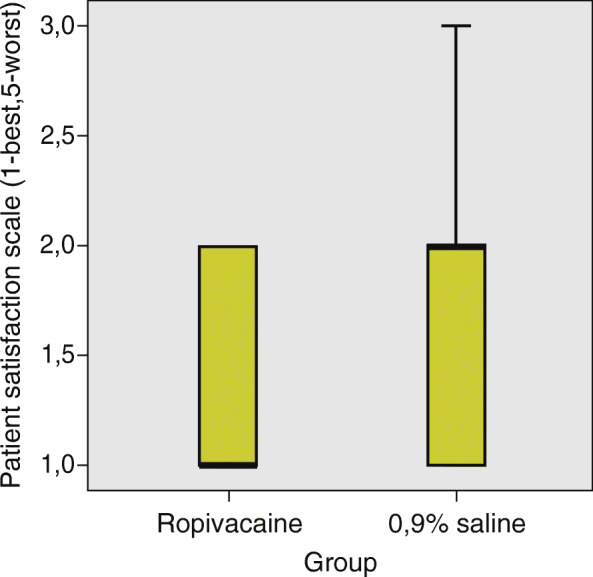
Comparison of patient satisfaction between the ropivacaine group and the 0,9% saline group

The median patient satisfaction with the pain relief measured by patient satisfaction scale ranging from 1-best to 5-worst, in the ropivacaine group was 1 (IQR 1). The median patient satisfaction with the pain relief in the 0.9% saline group was 2 (IQR 1). There was no significant difference between the two groups regarding pain relief satisfaction, Mann-Whitney U test, *p* = 0.130 (Fig. 5).

**Fig. 6:**
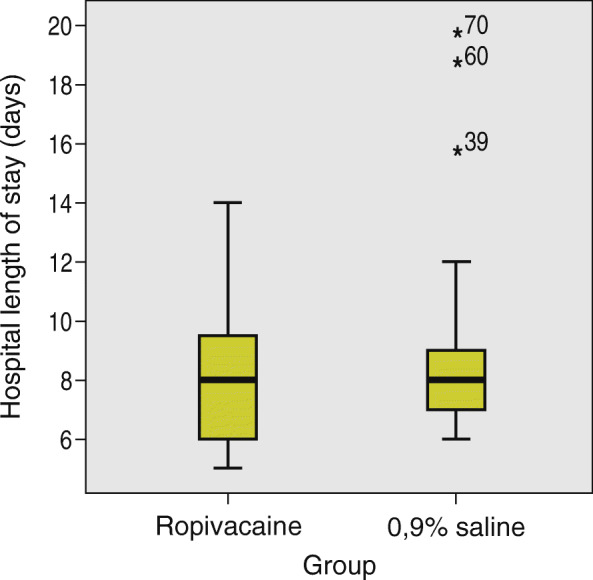
Comparison of hospital length of stay between the ropivacaine group and the 0,9% saline group

The median length of hospital stay in the ropivacaine group was 8 days (IQR 4 days). The median length of hospital stay in the 0.9% saline group was 8 days (IQR 2 days). There was no significant difference between the two groups in length of hospital stay, Mann-Whitney U test, *p* = 0.652 (Fig. 6).

There were no clinical signs of local anesthetic neurotoxicity nor cardiotoxicity in any one of the patients studied. No sample reached the maximum tolerated total plasma level of ropivacaine of 2.2 mg l^− 1^ as suggested by an earlier study on volunteers [23]. There were no clinical signs of wound infection nor delayed wound healing in any of the patients studied. All twenty tips of the wound catheters, (ten from each group) that were sent for microbiology analysis returned sterile.

## Discussion

The results of our study have shown that by administering a local anesthetic using a PCA pump there was a significant reduction of the opioid dose needed postoperatively. We also found significantly lower frequency of NRS scores higher than three in the ropivacaine group.

In recent decade, minimally invasive cardiac surgery has gained momentum in everday clincal practice mainly due to continuously growing medical technological innovations, progress in surgical techniques and advancement in anesthesiological experitse, involving modified patient monitoring, innovations in anesthesia drug delivery pathways, and utilization of short-acting anesthetics and multimodality in pain management.

Effective postoperative pain relief through multimodal approach is the goal every perioperative team aims to achieve. Improving postoperative analgesia leads to faster mobilizing, higher patient satisfaction and better surgical results [24, 25]. Even though the multimodal pain relief is central to most enhanced recovery protocols, it has been widely accepted that the treatment improvements need to be achieved at every step of the recovery process [2, 3], while at the same time also emphasizing the importance of adapting the protocols for each patient individually [26]. Using a PCA pump for delivering a local anesthetic in the surgical wound is a step closer to personalizing the multimodal analgesia to every patient. So far, most of the reports that studied wound infusion of local anesthetics have used an elastomeric pump as a delivery system [18, 27, 28]. To our knowledge, this is the first study that evaluates the effectiveness of PCA delivered local anesthetic at a minithoracotomy wound for AVR. Administering local anesthetic by PCA pump has certain advantages over an elastomeric pump. However, this concept assumes that the setting allows for an intensive care nurse or a physician to be available during the 48 h postoperatively to administer opioid i.v. when needed. The fact that the PCA pump stops delivering and alarms when the pressure in the catheter is too high is also a safety system. Elastomeric pump doesn’t stop administering, and what probably happens in these instances is that the local anesthetic is pushed through the more proximal holes of the catheter instead of flowing through all holes equally. This may lead to a less effective analgesia at the surgical wound, even more so when using longer wound catheters. Not least, some authors have reported cases of local anesthetic leaking back through the insertion point of the wound catheter [29], and there are also reports where local anesthetic wound infusion was of no benefit after full sternotomy [20, 29, 30]. The length of the wound catheter that has to be used in these patients combined with a delivery system without pressure alarm, may play a role. On the other hand using a PCA pump for delivering local anesthetic also allows the patient to administer a bolus, if needed. We had also encountered and had to overcome some technical difficulties as there is also a learning curve with correctly placing the catheter. During the pilot study we tested two ways of placing the wound catheter, one before wound closure and the other after the wound was closed. Placing the catheter before wound closure resulted in too many catheter occlusions probably due to the subcutaneous or the intradermal sutures causing the wound catheter to kink. We then realized that most patients undergoing AVR procedure, had only a thin muscle tissue if any, above the ribs II and III to serve as a cushion between the skin and the ribs which makes the catheter more likely to kink. Therefore inserting the wound catheter into the deep subcutaneous tissue and importantly after the wound was closed prevents kinking of the catheter at this sugical site. This way, we had no further technical difficulties with the catheter and the local anesthetic administration.

We did not observe any wound infections or impaired wound healing in this study. There were no signs of local anesthetic toxicity, based on continuous clinical assessment and monitoring. The patient satisfaction was similar in both groups, which proves that multimodal approach based on a local anesthetic wound infusion is not inferior to the more traditional i.v. opioid centered analgesia. However, our study did not show a shortened length of hospital stay for the local anesthetic group compared to the control group. This can be attributed to the fact that even the patients in the control group did not receive a large cumulative dose of opioid. We acknowledge that there was also the effect of the bolus of 0.75% ropivacaine that we administered to both groups at the end of the operation. It was done for ethical reasons as we were using an ultra short-acting opioid during the surgery. It is also important for the whole postoperative team (physiotherapists, nurses) to follow the advances in surgical minimally invasive techniques as certain rehabilitation procedures can be carried out earlier than after full sternotomy procedures. We argue that the minimally invasive cardiac surgery needs a separate recovery pathway from the classic cardiac surgery approach with full sternotomy. Other medical centers have already reported shorter length of hospital stay than in our study after minithoracotomy AVR [31].

We implemented the randomisaiton successfully, producing groups similary at baseline, thereby reducing the risk of selection bias. The use of placebo ensure avoidance of performance and measurement bias. It may be thought that there is a potential of bias when patients report their pain scores and satisfaction subjectively. However as this is randomized placebo-controlled study, the subjectivity probably influences patients in both groups the same way due to practitioner and patient blinding, thereby avoid the risk of bias arising in the main findings. Another limitation of our study is that patients in both groups received a bolus of 0.75% ropivacaine at the end of the operation in line with the ethical requirements for this study. Furthermore the multimodal protocol also included metamizole a non-opioid analgesic administered to patients in both groups regularly. Therefore, we may be critisized that baseline analgesic requirement and pain were relatively low, such that a prominent effect of continuous wound infiltration is hard to demonstrate. However, as we did show a significant difference this concern did not materialize in our study. Thus we are confident that our findings are valid and reliable. An additional limitation of our study is the fact that the surgery was carried by the same surgical team which has extensive experience in right anterior minithoracotomy approach. Although this is rather a strength of the procedure performed, it needs to be taken into consideration as it creates a steeper learning curve, when carrying out future analysis or incorporation into clinical practice. We also recognize some limitations in the generalisability as ours is a single center study.

Since our study, we have adopted the described pain relief protocol as a standard postoperative protocol for all our cardiac surgery patients when the procedure is performed with a minimally invasive technique. We use a wound catheter with PCA pump for all our minithoracotomy and ministernotomy AVRs, and also for our minithoracotomies for transapical TAVI procedures. The protocol is also made possible by our organisational structure which provides continuous monitoring of cardiac patients at least for the first 48 h, from ICU to HDU, where a nurse can check for pain levels hourly and administer an opioid bolus if needed.

## Conclusion

We found that wound infusion of local anesthetic by PCA pump significantly reduced opioid dose needed postoperatively. We have also shown that it is a feasible multimodal method of analgesia and it is in our opinion better suited to minimally invasive surgical techniques. Patient satisfaction with pain relief in our study was high. Therefore, we conclude that infusion of local anesthetic by PCA pump should be included in the multimodal pain control method following minimally invasive surgery.

## Data Availability

The datasets used and/or analysed during the current study are available from the corresponding author on reasonable request.

## Abbreviations

AVR: Aortic valve replacement
HDU: High dependency unit
ICU: Intensive care unit
NSAIDs: Nonsteroidal anti-inflammatory drugs
PCA: Patient controlled analgesia
RCT: Randomized controlled trial
NRS: Numeric rating scale
